# Comparisons of the biomechanical characteristics of the swing planes for the driver and 7-iron during the golf swing

**DOI:** 10.3389/fbioe.2026.1847830

**Published:** 2026-06-03

**Authors:** Heng Liu, Zilong Zhao, Chao Li, Chao Liu, Zefeng Wang, Baohua Liu

**Affiliations:** 1 School of Sports Training, Tianjin University of Sport, Tianjin, China; 2 Department of Social Sports, Hebei Sport University, Shijiazhuang, Hebel, China; 3 School of Social Sports, Tianjin University of Sport, Tianjin, China; 4 China Institute of Sport Science, General Administration of Sport of China, Dongcheng, Beijing, China

**Keywords:** different clubs, dynamics, golf, kinematics, swing plane

## Abstract

**Objective:**

To compare swing plane biomechanics between the driver and 7-iron in golf and elucidate the kinematic and dynamic mechanisms underlying swing plane formation.

**Methods:**

Sixteen male low-handicap golfers were recruited. A 3D motion capture system and two force plates were used to simultaneously record swing data for the driver and 7-iron. Swing plane angle, torso and pelvic angle, center of mass displacement, and ground reaction force (GRF) parameters were calculated. Statistical analyses were performed using paired t-tests, correlation analysis, and SPM {t}.

**Results:**

The driver showed a significantly smaller tilt angle than the 7-iron across all phases (p < 0.001), and a significantly smaller azimuth angle during the release-acceleration phase (p < 0.001). During the backswing, the 7-iron tilt angle was negatively correlated with torso rotation ROM (r = −0.701) and pelvic rotation ROM (r = −0.730), whereas the driver exhibited greater torso and pelvic rotation ROM than the 7-iron (both p < 0.05). During the release-acceleration phase, the driver tilt angle was positively correlated with pelvic forward tilt ROM (r = 0.850). SPM analysis further showed that the driver had a significantly smaller torso side tilt angle than the 7-iron during 0%–89% swing cycle (p = 0.001). During the downswing to follow through, the driver tilt angle was negatively correlated with AP RGRFmax (r = −0.805), whereas the 7-iron tilt angle was positively correlated with ML LGRFmax (r = 0.633). In addition, the driver exhibited significantly greater AP RGRF during 0%–5%, 37%–62%, and 83%–89% swing cycles, and significantly smaller ML LGRF during 0%–18% and 84%–93% swing cycles, compared with the 7-iron (p < 0.05).

**Conclusion:**

The driver relies on rotation of the torso and pelvis, combined with power generation from the rear, to create a flat swing plane, while the 7-iron maintains a steep and highly consistent plane through body posture control and front-side braking. Training should focus on strengthening the corresponding biomechanical capabilities to optimize the stability of swing plane.

## 1 Introduction

The golf swing is a typical multi-segment coordinated movement, with the ultimate goal of achieving precise and efficient energy transfer from body to the club and the ball (Shaw et al., 2023; Yang et al., 2024). The swing plane is defined as the best-fit reference plane derived from the shaft trajectory during the swing using singular value decomposition (SVD) (Coleman and Rankin, 2005; Morrison et al., 2018), representing the primary spatial orientation of the clubhead path rather than a literal planar movement trajectory. It is widely regarded by researchers as a key biomechanical factor influencing shot direction, distance, and consistency (Nasirzadeh et al., 2025; Corke et al., 2022; Chung, 2022). Specifically, swing plane tilt angle (inclination relative to vertical) regulates the steepness of the club path and launch condition, azimuth angle (horizontal orientation relative to the target line) determines the inside-out or outside-in club delivery direction, and root mean square error (RMSE) quantifies swing plane consistency and stability.

The driver and 7-iron serve functionally distinct purposes: the driver prioritizes maximum distance through high clubhead speed, whereas the 7-iron prioritizes precise shot control under varied lies and distances. Despite their distinct purposes, they are executed within the same fundamental swing system and kinetic chain in a given golfer, making direct within-subject comparison biomechanically meaningful. Such comparison can reveal how the motor system adaptively regulates swing mechanics to satisfy club-specific performance demands (Cheon et al., 2020). A fundamental question, however, remains unresolved: do swing plane differences between the driver and 7-iron reflect a scalable adjustment of a shared motor control strategy, or a qualitative reorganization of the kinetic chain? The distinction carries direct practical implications which scalable adjustments would permit cross-club training transfer, whereas kinetic-chain reorganization would necessitate club specific diagnosis and intervention. As the swing plane emerges from coordinated trunk motion, center of mass (COM) displacement, and ground reaction forces (GRF) (Shan et al., 2011), resolving this question requires full swing cycle kinematic and dynamic quantification. A within-subject design, in which the same golfers perform both clubs, inherently controls for anthropometry, strength, and coordination, thereby isolating the effect of club characteristics on swing plane regulation. To the best of our knowledge, such a full cycle, within-subject comparison has not yet been systematically reported.

Previous research on the swing plane has advanced along three converging lines. First, studies have defined the geometric features of the swing plane using motion capture and high-speed imaging. Khuyagbaatar et al. (2019) established a functional swing plane (FSP) from clubhead trajectories among low-handicap golfers and identified knee adduction and trunk rotation as key predictors of the X-factor (Khuyagbaatar et al., 2019). Coughlan et al. (2020) further demonstrated that longer-shafted clubs elicit flatter functional swing planes (Coughlan et al., 2020), consistent with the biomechanical demand that the driver’s longer shaft and greater clubhead mass require a shallower plane to lengthen the clubhead path and maximize linear velocity (Peterson and McNitt-Gray, 2019; Park and Seo, 2010). Kim and Park (2024) extended this framework by visualizing temporal convergence of swing-plane projections in the impact zone (Kim and Park, 2024). Collectively, these studies confirm that swing-plane morphology varies systematically across club types, yet remain largely descriptive and have not quantified the underlying kinematic and kinetic mechanisms that govern such differences. Second, research on the kinematic regulation of the swing plane has centered on torso pelvis coordination and the X-factor. Elite golfers exhibit greater pelvic rotation angles and peak angular velocities than amateurs, and actively decelerate the pelvis prior to impact to enhance distal energy transfer (Zhou et al., 2022). In male pro-golfers, pelvic rotation velocity during the downswing exceeds torso rotation velocity by 10%–15%, generating elastic potential energy that improves release efficiency (Lephart et al., 2007). The X-factor is widely recognized as a core component of the kinetic chain (Park and Seo, 2010; Gould et al., 2021; Gryc et al., 2023), and its peak value is a determinant of swing speed (Sidiropoulos et al., 2023). Nevertheless, these findings have been derived predominantly from a single club (typically the driver), and within-subject, inter-club comparisons remain scarce (McHugh et al., 2022; McHugh et al., 2024). Third, studies on the dynamic regulation of the swing plane have demonstrated the importance of COM displacement and GRF. Stable COM motion is critical for efficient force generation (Shan et al., 2011; İlhan Odabaş et al., 2019), and appropriate lateral and anterior COM shifts during the downswing create space for clubhead release (Morrison et al., 2018; Mahadas et al., 2019). Anterior–posterior GRF drives pelvic rotation toward the target (Parker et al., 2017; Liu et al., 2025; Kim et al., 2024), and vertical GRF is inversely coupled with vertical COM displacement across the backswing and downswing (Lynn et al., 2023; Brouwer et al., 2021). Yet COM and GRF have typically been treated as independent variables, and their integrated coupling with swing plane geometry has not been examined. Across all three domains, a pervasive methodological limitation persists: most studies rely on discrete, event-based data extracted at key swing milestones such as top of backswing or impact (Yang et al., 2024; İlhan Odabaş et al., 2019; Parker et al., 2017). Although such approaches have identified meaningful associations between peak X-factor, pelvic rotation at the top, COM position at impact, and swing performance, they inherently discard critical temporal information from transitional phases, including late backswing, transition, and early downswing, where swing plane geometry is actively modulated via rapid torso pelvis decoupling and dynamic GRF redistribution. Because driver and 7-iron swings diverge most profoundly in inter-segmental timing, sequencing, and phase lag within these critical windows, discrete event-based analyses risk failing to capture the core mechanisms of club-specific motor regulation. Two questions therefore remain unresolved: (Shaw et al., 2023): how do swing plane tilt angle, azimuth angle, and RMSE differ between the driver and 7-iron during the full swing, and at which phases are these differences most pronounced? (Yang et al., 2024) To what extent are these inter-club differences shaped by coordinated torso, pelvis motion and COM, GRF interactions rather than by isolated segmental adjustments? To address these questions, the present study combined SPM with specific phase of swing plane analysis and integrated kinematic and dynamic assessment. SPM was used to identify differences of club during normalized swing cycle, preserving temporal information that is lost in discrete event analyses. Evaluation of tilt, azimuth, and RMSE captured swing plane reorganization across the backswing, downswing, and follow-through. In addition, torso and pelvis ROM, X-factor, COM displacement, and GRF were analyzed together to characterize swing plane regulation within a unified biomechanical framework.

Accordingly, the present study aimed to compare the phases of swing plane characteristics between the driver and 7-iron during the normalized swing cycle using SPM, and to examine whether these differences were associated with torso and pelvis motion, COM displacement, and GRF patterns. We hypothesized that the two clubs would exhibit distinct swing plane geometries during the full swing, as reflected by differences in tilt angle, azimuth angle, and RMSE, with the largest differences occurring during the transition and early downswing phases. We further hypothesized that the driver would demonstrate greater torso and pelvis rotation ROM and X-factor than the 7-iron, whereas COM displacement and GRF would show specific patterns consistent with their different swing plane requirements. These hypotheses were formulated to be directly testable using the kinematic and dynamic variables assessed in the present study.

## 2 Subjects and methods

### 2.1 Subjects

Sixteen right-handed, low-handicap male golfers majoring in golf at Tianjin University of Sport were recruited for swing data collection (age: 20.75 ± 1.39 years; golf experience: 6.38 ± 2.60 years; handicap: 1.39 ± 2.06). An *a priori* sample size estimation was performed using G*Power 3.1.9.2 (Heinrich Heine University, Düsseldorf, Germany) based on the primary within-subject comparison of swing plane tilt angle between the driver and 7-iron. The expected effect size was derived from previous swing plane studies, and a conservative value of Cohen’s d = 0.80 was adopted (Kwon et al., 2012). With a two-tailed α = 0.05 and power (1-β) = 0.80, the minimum required sample size for the paired-samples t-test was n = 15. A sample of n = 16 was also considered sufficient to detect large bivariate correlations (|r|≥0.62) and to support SPM1d waveform analyses, consistent with prior biomechanical studies using comparable sample sizes (Gryc et al., 2023). Accordingly, 16 participants were recruited for the present study. Inclusion criteria were as follows: no major injury in the past 6 months, good physical condition during testing, and no strenuous exercise within 24 h before the experiment to ensure stable physical status (height: 1.76 ± 0.03 m, body mass: 76.18 ± 6.69 kg). This study was approved by the Ethics Committee of Tianjin University of Sport (No. TJUS 2025-111), and written informed consent was obtained from all participants prior to their involvement.

### 2.2 Testing protocol

**FIGURE 1:**
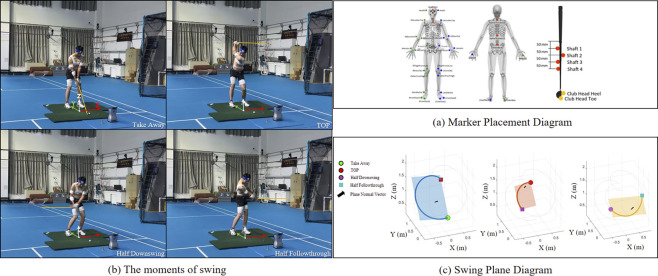
Maker Point Placement and Swing Definition. Note: Takeaway (TA) refers to the moment when clubhead speed reaches 0.1 mph; Top of the Backswing (TOP) refers to the moment when the club’s angular velocity reaches zero; Half-Downswing (HD) refers to the moment when the club is at the horizontal plane during its counterclockwise movement; Half Follow through (HF) refers to the moment when the club is at the horizontal plane after impact. **(a)** Marker Placement Diagram **(b)** The moments of swing **(c)** Swing Plane Diagram.

Participants used their own clubs and equipment that complied with the official rules of golf. To account for potential differences in club specifications among participants, the following parameters were verified for each participant’s driver and 7-iron prior to testing: shaft length (driver: 46.00 ± 0.18inch, 7-iron: 37.00 ± 0.25inch), shaft weight (driver: 72.88 ± 3.14g, 7-iron: 113.47 ± 13.44 g), shaft stiffness (driver: extra stiff flex of 11 and stiff flex of 5, 7-iron: extra stiff flex of 4 and stiff flex of 12) and loft angle (driver: 9.91° ± 0.73°, 7-iron: 34.72° ± 0.73°). Twelve Bridgestone-e12 balls were used as the test balls. Kinematic data were collected using a 16-camera motion capture system (Qualisys Track Manager, Qualisys AB, Sweden) with an infrared high-speed camera sampling rate of 250 Hz. Passive reflective markers were captured to obtain kinematic parameters during the driver and 7-iron swings (Figure 1a). GRF data were collected using two Kistler force plates (Model 9287C, dimensions: 900 mm × 600 mm × 100 mm; KISTLER, Switzerland) at a sampling rate of 1,000 Hz. An external amplifier was used to acquire GRF data, which were synchronised with the Qualisys motion capture system. A hitting cage measuring 3.15 m (height) × 1.6 m (width) × 2.8 m (length) was set up in the laboratory environment. Two golf mats were placed on top of the two force plates, with a distance of ≥4.5 m from the hitting cage.

All participants performed a 10 min standardized warm up while bare chest and wearing tight fitting sports pants, using their own golf clubs, gloves and shoes conforming to competition rules. The warm up consisted of dynamic stretching, practice swings and hitting drills to familiarize participants with the testing environment, prevent injury and optimize test performance. Following warm up, participants performed three additional swings with the first club (according to randomized order) to establish the baseline mean carry distance. These swings were conducted under conditions identical to formal trials. A baseline swing was considered successful if it met three criteria: (Shaw et al., 2023): complete execution without interruption; (Yang et al., 2024); clean ball contact with valid GCQuad high speed camera launch monitor (Foresight Sports, CA, United States) data; and (Coleman and Rankin, 2005) full-effort intent confirmed by the experimenter. Any unsuccessful swing was excluded and repeated until three successful swings were obtained. Carry distance from three swings were averaged to serve as participant specific and club specific baseline reference values. These baseline values were not included in the final biomechanical analysis but served solely as independent thresholds for trial validity screening (repeat the same procedure for the second club). Participants were instructed to stand on the hitting mat placed on the force plates. After confirming a stable setup posture, the experimenter gave a verbal cue to initiate data collection. Participants performed full golf swings according to their natural rhythm, and data collection was terminated by the experimenter upon completion of each swing (Figure 1b). The following standardized procedures were implemented: for the driver, the ball was positioned opposite the inner side of the lead heel on a 55 mm-height rubber tee. For the 7-iron, the ball was placed approximately 43 mm left of stance center. To guarantee consistency across trials and participants, chalk markings on the hitting mat fixed both ball placement and foot stance for every swing. The aiming direction was standardized perpendicular to the long axis of the force plates, with a visual target placed 4.5 m away at the center of the hitting cage. Testing order of the 7-iron and driver was randomized *via* a computer-generated permutation algorithm implemented in MATLAB R2021a (MathWorks, Natick, MA, United States). Participants were randomly assigned to one of two order conditions (the randperm function): driver-first (n = 8) or s7-iron-first (n = 8). The experimenter remained blinded to the sequence until immediately before each testing session to minimize potential bias. Five successful full swing trials were collected for each club, with an inter trial interval of 30 s and a 5 min rest period between clubs. All five valid trials were averaged for subsequent time-series and statistical analyses to reduce individual variability. After verifying complete data capture, swing validity was assessed using the GCQuad. A successful valid swing was defined by three pre-specified criteria: complete waveform data acquisition without signal interruption, carry distance within ±10% of the pre-established individual baseline mean carry distance, and directional deviation from the target line less than 5°. All trials meeting these predefined thresholds were retained for subsequent analysis.

### 2.3 Data processing

After the experiments, the raw collected data were pre-processed, including movement truncation and gap filling, and then exported in C3D format. All biomechanical calculations and processing, including filtering and interpolation, were performed using the Golf model in Visual3D software (C-motion Inc., MA, United States). The motion capture data were filtered using a bidirectional Butterworth low-pass filter with a cutoff frequency of 6 Hz, force plates data were filtered at 50 Hz and aligned according to the time stamps. A global coordinate system was defined with its origin at the point where the upper edges of the two force plates meet. The X-axis pointing the direction of the target (the direction of the ball’s flight), Y-axis pointing toward the body’s position, and Z-axis pointing vertically upward (Figure 1b). The COM was calculated as the mass center projected onto the horizontal plane based on a segmental model (Lenzi et al., 2003), and its displacement was decomposed into anterior-posterior (AP, anterior was positive), medio-lateral (ML, toward target was positive), and vertical (upward was positive) components. GRF data from the left and right force plates were independently processed and GRF values were normalized to body weight and expressed as % body weight (BW). AP-GRF defined to reaction force vector toward body was positive, ML-GRF defined to reaction force vector toward target was positive and vertical-GRF defined pushing upward was positive. GRFmax, the peak GRF value within each swing phase was extracted, LGRF refers to left foot and RGRF refers to right foot. Torso and pelvic orientations were defined using segment coordinate systems derived from the marker clusters. Torso and pelvis angles were calculated using Euler angles (X-Y-Z rotation sequence): Forward Tilt, the X-component of torso/pelvis rotation, positive when tilting forward (right-handed convention, same below). Side Tilt, the Y-component of torso/pelvis rotation, with leftward tilt being positive. Rotation, the Z-component of torso/pelvis rotation, with counterclockwise rotation being positive. X-Factor, the angle of torso rotation around the Z-axis relative to the pelvis, with counterclockwise rotation being positive.

Raw club data were exported and processed in MATLAB R2021a (MathWorks, MA, United States). A singular value decomposition (SVD) algorithm was implemented to fit the swing plane (Kwon et al., 2012; MacKenzie, 2012; Morrison and Wells, 2024) (Equations 1-5).

**FIGURE 2:**
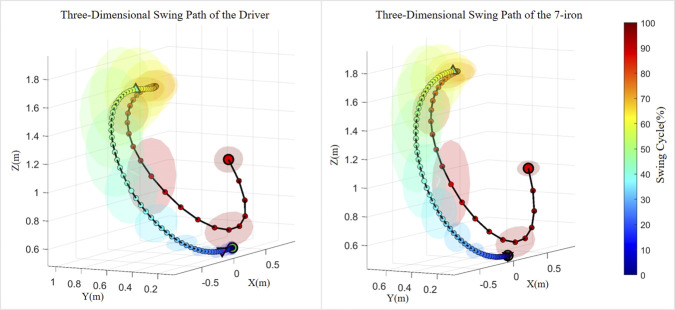
3D swing path diagram.

Select the midpoint of the marked points (Shaft1-4) on the shaft’s mid-section as the target point for plane fitting (Figure 2):

$$c=\frac{1}{n}\sum_{i=1}^{n}P_{i}$$ (1)

In the formula: c denotes the centroid of the data point.

$$A=\left[\begin{array}{c} P_{1}-c\\ P_{2}-c\\ \vdots \\ P_{n}-c \end{array}\right]$$ (2)

In the formula: A denotes the centered data matrix.

$$A=U\cdot \sum \cdot V^{T}$$ (3)

In the formula: Perform SVD plane solution on A , where U is an N×N left singular vector matrix, ∑ is an N×3 singular value diagonal matrix, and V is a 3×3 right singular vector matrix.

$$n=\left\{\begin{array}{c} v_{3}\;ifv_{3}\cdot e_{z}>0\\ -v_{3}\;otherwise \end{array}\right.$$ (4)

In the formula: n is the sign normalization factor ensuring consistent normal vector orientation (vertical component points toward the sky), $v_{3}$ is the swing plane normal vector, and $e_{z}$ is the vertical unit vector. After plane fitting, two core swing plane geometric parameters were mathematically derived from the normalized plane normal vector. Here, $e_{z}=\left[0,0,1\right]$ was defined as the vertical unit vector. The tilt angle quantified the inclination magnitude of the swing plane relative to the vertical Z-axis. A tilt angle of 0° represented a perfectly horizontal plane, while 90° indicated an entirely vertical plane. The azimuth angle described the horizontal orientation of the swing plane relative to the target line that angle ranged from −180° to 180°, where 0° denoted a swing plane perpendicular to the predefined target line. Positive azimuth values represented an out to inside swing path with the normal vector deviating rightward. The root mean square error (RMSE) was adopted to quantify the spatial deviation between phase-specific swing planes and evaluate intra-swing plane consistency. For each individual trial, the perpendicular distance $d_{i}$ between each downswing trajectory point and the corresponding backswing fitting plane was computed. RMSE formula was defined as:

$$RMSE=\sqrt{\frac{1}{N}\sum_{i=1}^{N}d_{i}^{2}\;}$$ (5)

In the formula: N represented the total number of discrete swing trajectory points. The lower the RMSE value, the higher the spatial overlap within swing phase.

### 2.4 Statistical analysis

Normality of the data was assessed using the Shapiro Wilk test. Paired sample t tests were used for normally distributed data, while the Wilcoxon Signed Rank Test was applied for non-normally distributed data. Correlation analyses between torso angle, pelvic angle, COM displacement, peak GRF, and swing plane angle at each phase were conducted using Pearson’s or Spearman’s correlation coefficients, as appropriate. False discovery rate (FDR) correction was applied to control for type I error inflation in multiple pairwise comparisons and correlation analyses. For time-series comparisons between driver and 7-iron, paired two-tailed SPM{t} analyses were conducted using the open-source SPM1D toolbox (m.0.4.10; https://spm1d.org/) in MATLAB R2021a. All time-series data were temporally normalized to 0%–100% swing cycle. Statistical thresholds were determined using random field theory (RFT) with an overall α = 0.05, providing corrected critical thresholds to account for multiple comparisons across the one-dimensional time series. Normality and residual assumptions underlying SPM1D were verified prior to formal analysis. Data are reported as mean ± standard deviation (M ± SD) for normally distributed variables or as median (interquartile range) [M50 (Q25, Q75)] for non-normally distributed variables. The significance level was set at p < 0.05.

## 3 Results

### 3.1 Swing plane angle

Backswing extends from the TA to the TOP; Release-acceleration Phase extends from the TOP to the HD; Downswing to the follow through extends from the HD to the HF (Figure 1c). As shown in Table 1, during the backswing, the tilt angle of the driver was significantly smaller than the 7-iron (p < 0.001), with no significant difference in the azimuth angle between the two clubs (p = 0.180). During the release and acceleration phase, both the tilt and azimuth angles of the driver were significantly smaller than the 7-iron (p < 0.001). During the downswing to follow through, the tilt angle of the driver remained significantly smaller than the 7-iron (p < 0.001), whereas no significant difference was found in the azimuth angle (p = 0.301). RMSE is a statistical measure reflecting the overall deviation between the measured data points and the theoretical fitted curve, indicating the average magnitude of the error. No significant differences in RMSE were observed between the two clubs across any swing phase (p > 0.05). Descriptively, both clubs exhibited higher RMSE values during the backswing (driver: 2.23 ± 0.81 cm vs. 7-iron: 1.74 ± 0.71 cm) compared to the downswing to follow through phase (driver: 0.53 ± 0.17 cm vs. 7-iron: 0.29 ± 0.12 cm).

**TABLE 1:** Swing plane parameters.

Phase	Swing plane angle	Driver	7-Iron	t/z	p
Backswing	Tilt angle (°)^a^	60.49 ± 3.63	70.61 ± 3.56	−22.997	<0.001
	Azimuth angle (°)	−5.35 ± 6.43	−4.29 ± 5.82	−1.407	0.180
	RMSE (cm)	2.23 ± 0.81	1.74 ± 0.71	1.732	0.200
Release-acceleration phase	Tilt angle (°)^a^	50.44 (46.89, 52.41)	61.89 (58.52, 64.47)	−3.516	<0.001
	Azimuth angle (°)^a^	−22.50 ± 10.01	−17.95 ± 9.03	−6.633	<0.001
	RMSE (cm)	1.09 ± 0.47	0.88 ± 0.47	1.375	0.527
Downswing to the follow through	Tilt angle (°)^a^	48.86 (48.22, 50.33)	63.69 (60.10, 64.90)	−3.516	<0.001
	Azimuth angle (°)	−1.58 (-4.55, 4.68)	0.09 (-3.00, 6.79)	−1.034	0.301
	RMSE (cm)	0.53 ± 0.17	0.29 ± 0.12	1.449	0.441

^a^indicates a significant difference between the driver and 7-iron (p < 0.05), the same below.

### 3.2 Torso and pelvis analysis

During the backswing, the driver exhibited significantly greater torso rotation ROM and pelvic rotation ROM compared to the 7-iron (p < 0.05), while the driver’s torso forward tilt ROM and torso side tilt ROM were significantly smaller than the 7-iron (p < 0.05). No significant differences were found in pelvic forward tilt ROM or pelvic side tilt ROM between the two clubs (p > 0.05). During the release-acceleration phase, the driver showed significantly greater torso rotation ROM, pelvic forward tilt ROM, pelvic side tilt ROM, and pelvic rotation ROM than the 7-iron (p < 0.05). In contrast, the driver’s torso forward tilt ROM was significantly smaller than the 7-iron (p < 0.001), while no significant difference was observed in torso side tilt ROM (p > 0.05). During the downswing to follow through, the driver demonstrated significantly greater torso forward tilt ROM and torso rotation ROM compared to the 7-iron (p < 0.05). However, the driver’s torso side tilt ROM, pelvic forward tilt ROM, and pelvic side tilt ROM were significantly smaller than the 7-iron (p < 0.05), with no significant difference in pelvic rotation ROM (p > 0.05). The detailed torso and pelvic joints parameters were presented in Table 2.

**TABLE 2:** ROM of torso and pelvic joints.

ROM (°)	Backswing		Release-acceleration phase		Downswing to the follow through	
	Driver	7-Iron	Driver	7-Iron	Driver	7-Iron
Forward tilt-torso	40.50 ± 5.53*	46.13 ± 4.86	45.91 ± 6.89*	52.63 ± 9.99	40.20 ± 8.80*	34.58 ± 6.21
Side tilt-torso	51.72 ± 8.90*	56.11 ± 7.53	47.02 ± 7.86	48.97 ± 6.03	32.48 ± 9.11*	42.00 ± 7.71
Rotation-torso	111.83 ± 11.78*	103.88 ± 11.41	103.58 ± 12.35*	94.57 ± 13.74	62.72 (59.39, 68.25)*	52.99 (50.30, 61.94)
Forward tilt-pelvis	7.80 (7.28, 8.56)	8.91 (6.99, 11.93)	12.50 ± 3.23*	9.33 ± 3.31	8.67 ± 3.16*	9.47 ± 3.24
Side tilt-pelvis	18.86 (14.93, 20.78)	18.90 (13.48, 20.69)	24.00 ± 2.83*	22.91 ± 4.42	5.26 (2.96, 7.47)*	6.62 (4.50, 8.49)
Rotation-pelvis	58.64 ± 8.70*	52.27 ± 7.09	86.02 ± 10.54*	73.82 ± 10.02	29.53 (21.17, 35.48)	27.64 (24.38, 34.28)

ROM, was defined as the angular difference between the maximal and minimal values within each swing phase.

**FIGURE 3:**
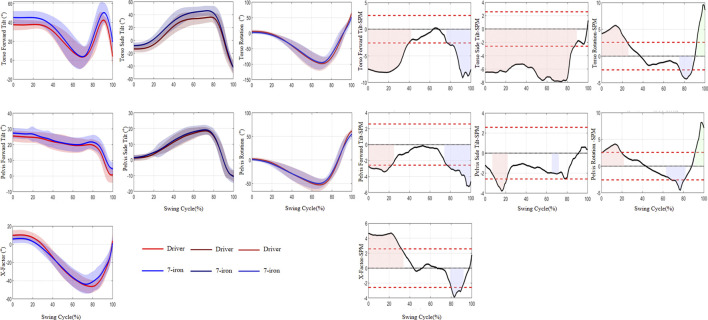
SPM analysis of torso and pelvic angles.

The normalized swing phases were defined as follows: backswing (Driver: 0%–73% vs. 7-iron: 0%–72%), release-acceleration phase (Driver: 73%–91% vs. 7-iron: 72%–91%), and downswing to the follow through (both 91%–100%). As shown in Figure 3, the driver exhibited significantly smaller torso forward tilt angles than the 7-iron during 0%–47% (p = 0.007) and 76%–100% (p = 0.002) swing cycle. Torso side tilt angles for the driver were significantly smaller than the 7-iron during 0%–89% (p = 0.001) swing cycle. For torso rotation, the driver showed significantly greater angles during 0%–26% (p = 0.002) and 92%–100% (p = 0.001) swing cycle, but significantly smaller angles during 75%–88% (p = 0.005) compared to the 7-iron. The driver exhibited significantly smaller pelvic forward tilt angles than the 7-iron during 0%–25% (p = 0.005) and 74%–100% (p = 0.004) swing cycle. The driver showed significantly smaller pelvic side tilt angles than the 7-iron during 7%–22% (p = 0.009) and 65%–72% (p = 0.045) swing cycle. For pelvic rotation, the driver showed significantly greater angles during 0%–22% (p = 0.006) and 91%–100% (p = 0.003) swing cycle, but significantly smaller angles during 63%–82% (p = 0.013) compared to the 7-iron. X-Factor for the driver was significantly greater than the 7-iron during 0%–34% (p = 0.002) swing cycle, but significantly smaller during 79%–92% (p = 0.009) swing cycle.

### 3.3 COM and GRF analysis

During the backswing, the driver exhibited significantly greater ML ΔCOM, ML LGRFmax, AP RGRFmax, and vertical RGRFmax compared to the 7-iron (all p < 0.05). The driver’s vertical ΔCOM was significantly smaller than the 7-iron (p = 0.003). No significant differences were observed in AP ΔCOM or AP LGRFmax (p > 0.05, Table 3). During the release-acceleration phase, the driver showed significantly greater AP RGRFmax and vertical RGRFmax than the 7-iron (p < 0.05). In contrast, the driver’s AP ΔCOM and AP LGRFmax were significantly smaller than the 7-iron (p < 0.05). No significant differences were found in ML ΔCOM, vertical ΔCOM, ML LGRFmax, vertical LGRFmax, or ML RGRFmax (p > 0.05). During the downswing to the follow through, the driver demonstrated significantly greater ML LGRFmax and vertical RGRFmax than the 7-iron (p < 0.05), while ML ΔCOM, AP ΔCOM, and AP RGRFmax were significantly smaller (p < 0.05). No significant differences were observed in vertical ΔCOM, AP LGRFmax, vertical LGRFmax, or ML RGRFmax (p > 0.05).

**TABLE 3:** Changes in COM displacement (cm) and Peak GRF (BW) during various phases.

COM and GRF indicators	Backswing		Release-acceleration phase		Downswing to the follow through	
	Driver	7-Iron	Driver	7-Iron	Driver	7-Iron
ML Δ COM	−1.80 ± 2.08*	−0.33 ± 2.05	5.60 (2.28, 7.41)	4.52 (3.56, 7.32)	0.75 (-0.61, 1.83)*	1.75 (1.35, 2.82)
AP Δ COM	−1.12 (-2.67, 0.02)	−0.62 (-2.28, 0.36)	0.75 (0.41, 1.88)*	1.43 (0.58, 1.80)	−0.43 (-1.16, 0.44)*	0.09 (-0.53, 0.45)
Vertical Δ COM	1.54 (0.58, 2.56)*	2.18 (1.00, 3.46)	−4.37 (-5.16, 0.93)	−3.40 (-5.66.-0.62))	3.70 (-0.64, 6.36)	2.11 (-0.42, 4.54)
ML LGRFmax	−0.01 ± 0.03	−0.02 ± 0.03	0.02 ± 0.03	0.02 ± 0.02	0.00 ± 0.04*	−0.03 ± 0.04
AP LGRFmax	0.07 ± 0.02	0.07 ± 0.02	−0.04 ± 0.04*	−0.02 ± 0.04	0.10 ± 0.02	0.09 ± 0.02
Vertical LGRFmax	0.57 ± 0.08	0.56 ± 0.09	1.27 ± 0.22	1.20 ± 0.31	1.17 ± 0.27	1.21 ± 0.32
ML RGRFmax	0.15 ± 0.02*	0.12 ± 0.01	0.15 (0.10, 0.20)	0.12 (0.10, 0.18)	0.11 (0.09, 0.21)	0.12 (0.11.0.17)
AP RGRFmax	0.10 (0.05, 0.13)*	0.08 (0.01, 0.09)	0.17 (0.16, 0.21)*	0.16 (0.14, 0.19)	0.06 ± 0.04*	0.08 ± 0.03
Vertical RGRFmax	0.82 (0.75, 0.97)*	0.83 (0.77, 0.93)	0.67 ± 0.15*	0.56 ± 0.13	0.72 ± 0.26*	0.62 ± 0.24

ΔCOM, refers to the displacement of the COM, during the swing, specifically the vector difference between the final and initial positions of the COM.

**FIGURE 4:**
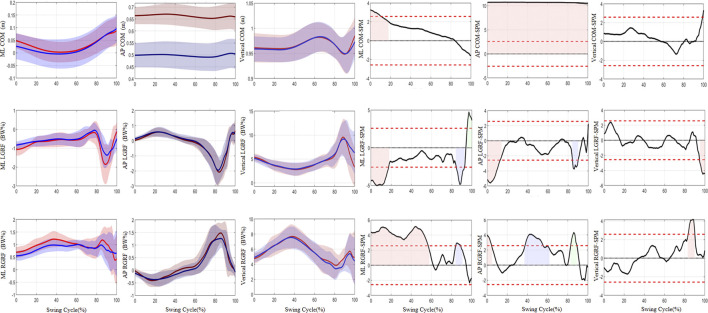
COM and GRF SPM analysis.

As shown in Figure 4, the ML COM displacement of driver was significantly greater than the 7-iron during 0%–18% swing cycle (p = 0.002). AP COM displacement for the driver was significantly greater than the 7 iron across 0%–100% swing cycle (p < 0.001). No significant difference was found in vertical COM displacement across 0%–100% swing cycle (p > 0.05). For ML LGRF, the driver showed significantly smaller than 7-iron during 0%–18% (p = 0.002) and 84%–93% (p = 0.006) swing cycle, but significantly greater than 7-iron during 95%–100% (p = 0.008) swing cycle. The driver exhibited significantly smaller AP LGRF than the 7-iron during 0%–14% (p = 0.004) and 84%–90% (p = 0.007) swing cycle. Vertical LGRF for the driver was significantly smaller than the 7-iron during 95%–100% (p = 0.001) swing cycle. The driver exhibited significantly greater ML RGRF than the 7-iron during 0%–58% (p = 0.011) and 84%–90% (p = 0.015) swing cycle. The driver exhibited significantly greater AP RGRF than the 7-iron during 0%–5% (p = 0.004), 37%–62% (p = 0.007) and 83%–89% (p = 0.003) swing cycle. Vertical RGRF for the driver was significantly greater than the 7-iron during 83%–90% (p = 0.047) swing cycle.

### 3.4 Correlation analysis of swing plane angles

#### 3.4.1 Backswing

For the driver, no correlations met the |r|≥0.6 threshold. For the 7-iron, the tilt angle was significantly negatively correlated with torso rotation ROM (r = −0.701), pelvic rotation ROM (r = −0.730), AP LGRFmax (r = −0.814), vertical LGRFmax (r = −0.613), and AP RGRFmax (r = −0.657) (all p < 0.05).

#### 3.4.2 Release-acceleration phase

For the driver, the tilt angle was significantly positively correlated with pelvic forward tilt ROM (r = 0.850, p < 0.001), while the azimuth angle was significantly negatively correlated with torso forward tilt ROM (r = −0.603, p = 0.013). For the 7-iron, the tilt angle was significantly positively correlated with vertical COM displacement (r = 0.850, p < 0.001), and significantly negatively correlated with torso rotation ROM (r = −0.690) and AP ΔCOM (r = −0.899) (all p < 0.05). The azimuth angle of the 7-iron was significantly negatively correlated with torso forward tilt ROM (r = −0.679), torso rotation ROM (r = −0.618), pelvic rotation ROM (r = −0.607), and AP ΔCOM (r = −0.886) (all p < 0.05).

#### 3.4.3 Downswing to the follow through

**FIGURE 5:**
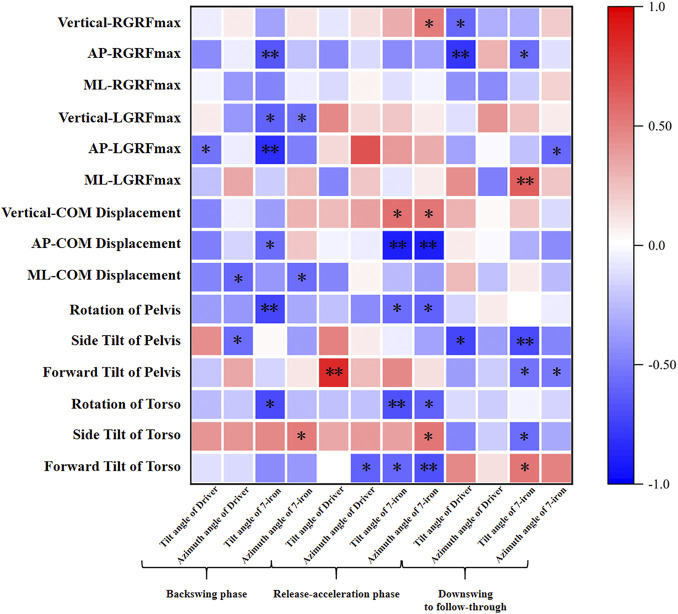
Correlation Heat Map. Note: Only correlations with |r|≥0.6 are reported below, consistent with the statistical power analysis.

For the driver, the tilt angle was significantly negatively correlated with pelvic side tilt ROM (r = −0.719) and AP RGRFmax (r = −0.805) (both p < 0.05). For the 7-iron, the tilt angle was significantly positively correlated with ML LGRFmax (r = 0.633, p = 0.008), and significantly negatively correlated with pelvic side tilt ROM (r = −0.692, p = 0.003). Other results below the threshold of |r|≥0.6 are not interpreted; non-significant correlations are presented in Figure 5.

## 4 Discussion

The golf swing relies on coordinated multi-segment motion, wherein technical optimisation requires clarifying the associations between club specifications, human movement patterns, and mechanical loading behaviours (Bourgain et al., 2018; Nesbit and McGinnis, 2009). While previous research has primarily described swing plane and kinematic parameters for a single club (Khuyagbaatar et al., 2019; Bourgain et al., 2022) few studies have systematically compared driver and 7-iron swing biomechanics during full swing within the same golfers, and an integrated analysis of swing plane, kinematics, and dynamics has not yet been reported. Unlike previous studies, the present study performed a within-subject comparative analysis to systematically explore club dependent discrepancies between the driver and 7-iron. This study synchronously quantified swing plane geometry (tilt angle, azimuth angle and RMSE), torso and pelvis kinematics, COM displacement, and GRF characteristics during the swing cycle. Notably, the analytical approaches adopted in this work were limited to inter-group comparison and correlation analysis. Accordingly, all observed relationships are interpreted as statistical associations rather than causal mechanical pathways. These observational findings objectively describe phase biomechanical distinctions between two clubs, providing empirical data for understanding swing regulation in low-handicap golfers. Regarding the phase division of the driver and 7-iron swings, the present study found that only during the backswing phase did the driver show a 1% shorter duration than the 7-iron, while the remaining phases were highly consistent between the two clubs. The driver exhibited a smaller tilt angle, which is statistically consistent with the theory that longer-shafted clubs require a flatter swing trajectory to generate higher clubhead speed (Mackenzie et al., 2018; Wallace et al., 2007). During the release-acceleration phase, the driver not only had a smaller tilt angle but also a significantly more negative azimuth angle than the 7-iron (−22.50 ± 10.01°vs. −17.95° ± 9.03°, p < 0.001). As defined in Methods negative values indicating an inside to out path. The observed more negative azimuth angle for the driver therefore corresponds to a more pronounced inside to out path orientation during this phase. Based on previous mechanical theories, such an inside to out swing pattern is considered conducive to facilitating whipping motion and restraining excessive lateral clubhead deviation (Zhang and Shan, 2014). However, the present study did not directly measure kinetic chain sequencing, clubhead speed, or distance loss; therefore, whether the observed more negative azimuth angle in the driver translates into enhanced energy transfer efficiency remains to be empirically tested in future studies. No statistically significant differences in RMSE were observed between the driver and 7-iron across any swing phase (Table 1). The driver’s backswing RMSE was 2.23 ± 0.81cm, while during the downswing to follow through, RMSE values for both clubs were below 1 cm (driver: 0.53 ± 0.17cm; 7-iron: 0.29 ± 0.12 cm). The numerically higher backswing RMSE for the driver is reported as a descriptive observation rather than a statistically tested between club differences. Previous studies have reported that planar fitting quality can vary across swing phases and may be influenced by club length (Coleman and Rankin, 2005; Kwon et al., 2012). However, no validated thresholds currently exist for interpreting RMSE values in golf swing plane analysis, and these descriptive differences require further contextual clarification in future mechanical verification.

The torso and pelvis serve as the central hubs of the golf swing kinetic chain (Kim, 2025; Lamb and Pataky, 2018), and their kinematic differences determine the divergence in swing plane characteristics. Club kinematic disparities are mechanically driven by inherent differences in shaft length and inertial properties; longer-shafted clubs require greater rotational trunk mobility to overcome inertial resistance (Yang et al., 2024). During the backswing, the driver exhibited significantly greater torso rotation ROM and pelvic rotation ROM than the 7-iron, while its tilt angle was significantly smaller. This finding is consistent with Zheng et al. (2008), but extends their work by demonstrating similar rotational differences in a within-subject design, thereby reducing inter-individual variability. For the 7-iron, the tilt angle was significantly negatively correlated with both torso rotation ROM and pelvic rotation ROM, indicating that smaller rotational amplitudes of the torso and pelvis were associated with a steeper swing plane. This relationship mechanically originates from the low inertial resistance of the short-shafted iron, which may allow lateral torso tilt to geometrically override horizontal rotation and steepen the swing plane (Morrison and Wells, 2024). These findings also confirmed that the driver adopts a rotation dominated strategy during the backswing, establishing torsional reserves for subsequent energy release by increasing horizontal rotation. During the release-acceleration phase, the driver continued to show significantly greater torso rotation ROM and pelvic rotation ROM than the 7-iron, while both its tilt and azimuth angles remained significantly smaller. The driver’s pelvic forward tilt ROM was positively correlated with its tilt angle, whereas for the 7-iron, the tilt angle was negatively correlated with torso rotation ROM and AP ΔCOM, and the azimuth angle was negatively correlated with torso forward tilt ROM and AP ΔCOM. These correlation patterns represent a novel finding. These divergent correlation patterns may reflect two distinct mechanical regulation models: the driver relies on sagittal pelvic tilt to modulate a shallow plane, the iron coordinates anteroposterior mass transfer and torso inclination to adjust a steeper delivery path. While previous study showed that the X-factor is related to clubhead speed (Gould et al., 2021), it did not examine how torso-pelvis kinematic couplings regulate swing plane across different clubs. Our results indicate that swing plane angle formation during the release-acceleration phase depends not only on rotational range, but also on anterior-posterior weight shift and torso forward tilt control. During the downswing to follow through, pelvic side tilt ROM was significantly smaller for the driver than for the 7-iron, and the tilt angle was negatively correlated with pelvic side tilt ROM in both clubs, suggesting that pelvic side tilt is an important factor regulating plane tile angle in this phase. A key methodological advance of this study is the use of SPM, which identified continuous between club differences during the downswing to follow through, including torso side tilt angle (0%–89%) and pelvic rotation angle (63%–82%). These phase specific effects would likely be missed by traditional discrete event analyses (Zhou et al., 2022; Zheng et al., 2008). The long duration torso side tilt difference across nearly the full swing cycle aligns with persistent inter-club lateral postural modulation. Notably, the pelvic rotation difference coincided with the release-acceleration phase, where swing-plane azimuth differences were also observed, suggesting a direct phase-specific regulatory role of pelvic rotation in swing-plane azimuth control. During the release-acceleration phase, the X-factor reached its minimum value, with the pelvis initiating a reverse rotation first, allowing elastic potential energy to accumulate to its peak and then be rapidly converted into clubhead kinetic energy (Gould et al., 2021). The 7-iron exhibited an opposite kinematic trend: a smaller X-factor in the early swing phase enhanced swing stability (Yang et al., 2024; Dale and Brumitt, 2016), while a larger X-factor in the later swing phase provided space for swing path adjustments.

The COM and GRF results further indicate that the differences in swing plane between the two clubs are also influenced by the patterns of weight transfer and ground force application. All significant COM-GRF correlations observed in the present study likely represent structured force motion relationships that support dynamic postural equilibrium under different inertial loads. During the backswing, the driver exhibited significantly greater ML ΔCOM, ML LGRFmax, AP RGRFmax, and vertical RGRFmax than the 7-iron, while its vertical ΔCOM was significantly smaller. Furthermore, the tilt angle of 7-iron was negatively correlated with AP LGRFmax, and AP RGRFmax. To achieve a smaller tilt angle and maintain an upward impact orientation, the driver setup requires more right-side trunk lateral bending (Parker et al., 2017; Kim et al., 2023). Therefore, during the backswing, the driver demands greater right side lateral GRF to maintain body posture compared to the 7-iron. Mechanically, the increased lateral bending and unilateral right side force generation may serve to counterbalance the higher inertial torque of the longer driver shaft (Mackenzie et al., 2018). During the release-acceleration phase, the driver’s AP RGRFmax and vertical RGRFmax were significantly greater than the 7-iron, while its AP ΔCOM was significantly smaller. This indicates that the driver relies on explosive anterior-posterior and vertical forces from the right foot to drive energy release, with a more concentrated COM displacement. In contrast, the 7-iron adopts an opposite COM movement strategy. During the downswing to follow through, the driver’s AP RGRFmax, ML ΔCOM, and AP ΔCOM were all significantly smaller than the 7-iron. Additionally, for the 7-iron, the tilt angle was positively correlated with ML LGRFmax, while for the driver, the tilt angle was negatively correlated with AP RGRFmax. Collectively, these findings imply that swing-plane regulation is club specific and depends on distinct GRF strategies: the driver emphasizes rotational stability and restrained COM shift, whereas the 7-iron depends more on lead-foot lateral support to maintain tilt and control swing geometry. Previous studies have found that to achieve a smaller swing plane inclination angle, the driver setup involves greater trunk forward tilt compared to irons (Park and Seo, 2010). However, in the present study, the driver showed significantly smaller torso forward tilt ROM than the 7-iron during the backswing (driver: 40.50° ± 5.53° vs. 7-iron: 46.13° ± 4.86°, p < 0.05) and release-acceleration (driver: 45.91° ± 6.89° vs. 7-iron: 52.63° ± 9.99°, p < 0.05) phases, and the SPM analysis indicated a significantly smaller torso forward tilt angle for the driver during 0%–47% and 76%–100% swing cycle. The early interval (0%–47%) reflects a relatively upright static setup posture, the late interval (76%–100%) shows sustained upright trunk kinematics during inertial motion. Although the driver may require greater torso forward tilt at address, the phases data showed a more upright torso posture for the driver over much of the swing. Thus, the AP COM differences between clubs (Figure 4) likely reflect both the initial setup posture and torso kinematics, rather than a consistently greater torso forward tilt during the swing (Ball and Best, 2007; Souaifi et al., 2025). This suggests that COM regulation in the golf swing is shaped by the combined effects of static address position and dynamic postural adjustments. The SPM analysis of GRF waveforms provides additional insight unavailable from discrete peak comparisons alone. The driver exhibited significantly larger ML RGRF during 0%–58% and 84%–91% swing cycle, and significantly larger AP RGRF during 0%–5%, 37%–62%, and 83%–89% swing cycle compared to the 7-iron. These prolonged right limb dominant force intervals overlap with backswing stabilization and acceleration energy generation stages, and this temporal pattern is associatively consistent with continuous right lower limb loading for momentum generation. In contrast, the 7-iron showed significantly larger ML LGRF during 0%–18% and 84%–93%, AP LGRF during 0%–14% and 84%–90%, and vertical LGRF during 95%–100% swing cycle compared to the driver, suggesting that it places greater emphasis on left lower-limb support and braking. These temporally localized differences reveal that GRF patterns are not uniformly distributed during the swing but are concentrated in specific phases where each club’s unique mechanical demands are most pronounced. Traditional peak-based comparisons would have obscured this temporal structure (Shan et al., 2011; Choi et al., 2016), which provides descriptive insight into when during the swing each lower limb contributes to club-specific regulation. Crucially, these temporally localized differences are reported as descriptive associations and the functional significance of these time-specific patterns remains to be tested in future studies.

Several limitations of this study should be acknowledged. All participants were low-handicap male golfers, which limits the representativeness of the sample. Meanwhile, each golfer used their own personal clubs, leading to uncontrolled variations in club specifications; such equipment heterogeneity may inevitably introduce confounding biomechanical variations. Future studies should include players of different skill levels or both sexes to compare swing plane characteristics when using different clubs and should consider using standardized club sets across participants to minimize equipment-related variability. Although the laboratory environment provided precise measurements, it may not fully replicate the conditions and constraints of actual course play. This study did not examine the coupling between shot outcome and swing plane parameters, and therefore could not determine the relative contribution of swing plane parameters to shot performance. In future research, golfers of varying skill levels and swing styles should be included to investigate individual differences in the swing plane, thereby providing a basis for personalised training interventions.

## 5 Conclusion

The driver overall exhibits a flatter swing trajectory and a more pronounced inside to out path during the release-acceleration phase. Meanwhile, the larger RMSE of the driver during the backswing indicates stronger non-planarity of its trajectory, whereas swing trajectories of both clubs tend to be planarized with more stable movement control during the downswing to follow through. During backswing, the driver builds torsional reserves through large amplitude rotation of the torso and pelvis to form a shallower swing plane. The 7-iron forms a steeper swing plane *via* greater control of torso forward tilt and side tilt posture. During the release-acceleration phase, the driver relies mainly on pelvic forward tilt regulation and multi directional force generation from the trail lower limb to reduce plane incline angle and azimuth angle, resulting in a more inside to out swing path. The 7-iron maintains plane stability through torso side tilt, vertical COM displacement, and lead lower limb support. During the downswing to follow through, the driver reduces pelvic lateral bending and utilises rear-leg vertical support to maintain a shallower plane and complete inertial release, whereas the 7-iron employs torso forward tilt, pelvic side tilt, front-leg support and braking to ensure a larger tilt angle and stable shot direction. In practice, training for the driver should emphasise torso-pelvis separation, rear-leg push-off, and weight transfer control to stabilise the shallow swing plane and increase swing speed. Training for the 7-iron should focus on trunk posture regulation and front-leg support and braking to maintain plane consistency and improve shot accuracy.

## Data Availability

The original contributions presented in the study are included in the article/supplementary material, further inquiries can be directed to the corresponding author.
